# Multisystem Langerhans cell histiocytosis in an adult non‐smoker treated with steroid therapy

**DOI:** 10.1002/rcr2.603

**Published:** 2020-06-12

**Authors:** Haruka Ito, Masaru Ito, Yukio Kakuta, Takeshi Kaneko, Koji Okudera, Takashi Ogura

**Affiliations:** ^1^ Department of Respiratory Medicine Yokohama Rosai Hospital Yokohama Kanagawa Japan; ^2^ Department of Pathology Yokohama Rosai Hospital Yokohama Kanagawa Japan; ^3^ Department of Pulmonology Yokohama City University Graduate School of Medicine Yokohama Kanagawa Japan; ^4^ Department of Pathology Kanagawa Cardiovascular and Respiratory Center Yokohama Kanagawa Japan; ^5^ Department of Respiratory Medicine Kanagawa Cardiovascular and Respiratory Center Yokohama Kanagawa Japan

**Keywords:** Corticosteroid therapy, Langerhans cell histiocytosis, non‐smoker, transbronchial lung cryobiopsy

## Abstract

We describe the case of a 29‐year‐old female non‐smoker who was treated with steroid therapy for a subacute exacerbation of multisystem Langerhans cell histiocytosis (MS‐LCH) with worsening lung, skin, and oral mucosal lesions. The patient developed pneumonia, and computed tomography (CT) showed multiple thin‐walled cavities. Transbronchial lung cryobiopsy (TBLC) specimens revealed Langerhans cells, which were positive for CD1a and S‐100 expression. Similar histological findings were detected in the submandibular gland, skin, and tooth. On the basis of these findings, the patient was diagnosed with MS‐LCH and subsequently treated with steroid therapy. From the literature review, case reports of non‐smokers with pulmonary lesions that worsened and required treatment are rare. Almost all cases recurred and needed additional treatment. This case study contributes to our understanding of the potential role of steroid therapy in MS‐LCH treatment. Additionally, TBLC is a novel, potentially safer, diagnostic tool that has not been previously described for LCH.

## Graphical abstract

Multisystem Langerhans cell histiocytosis in an adult non‐smoker treated with steroid therapy for a subacute exacerbation: a case report and review of the literature.

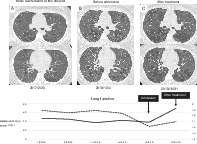

## Introduction

Langerhans cell histiocytosis (LCH) is a rare disease of clonal dendritic cells, which may affect any organ of the body. It frequently involves the skeleton (80% of cases), skin (33%), and pituitary (25%). Other involved organs include the spleen, haematopoietic system, lungs (15% each), lymph nodes (5–10%), and the central nervous system excluding the pituitary (2–4%) [1]. Additionally, pulmonary LCH (PLCH) is associated with smoking [2]. Herein, we report a rare case of multisystem LCH (MS‐LCH) in an adult non‐smoker diagnosed by transbronchial lung cryobiopsy (TBLC). In this patient, MS‐LCH infiltrated the lung, pituitary, submandibular gland, skin, and tooth.

## Case Report

**Figure 1:**
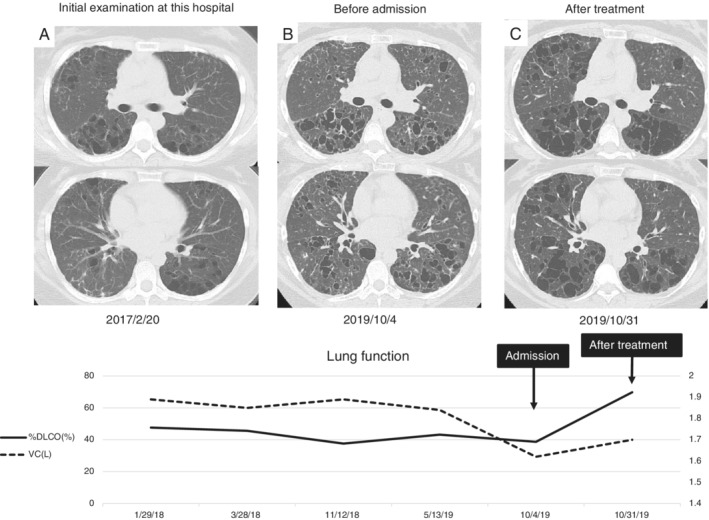
Computed tomography (CT) images and lung function in the patient's clinical course. CT findings revealed multiple thin‐walled cavities and nodules (A–C). These findings worsened in three months. The patient's lung function worsened before admission. After steroid therapy, the patient's CT findings and lung function improved.

**Figure 2:**
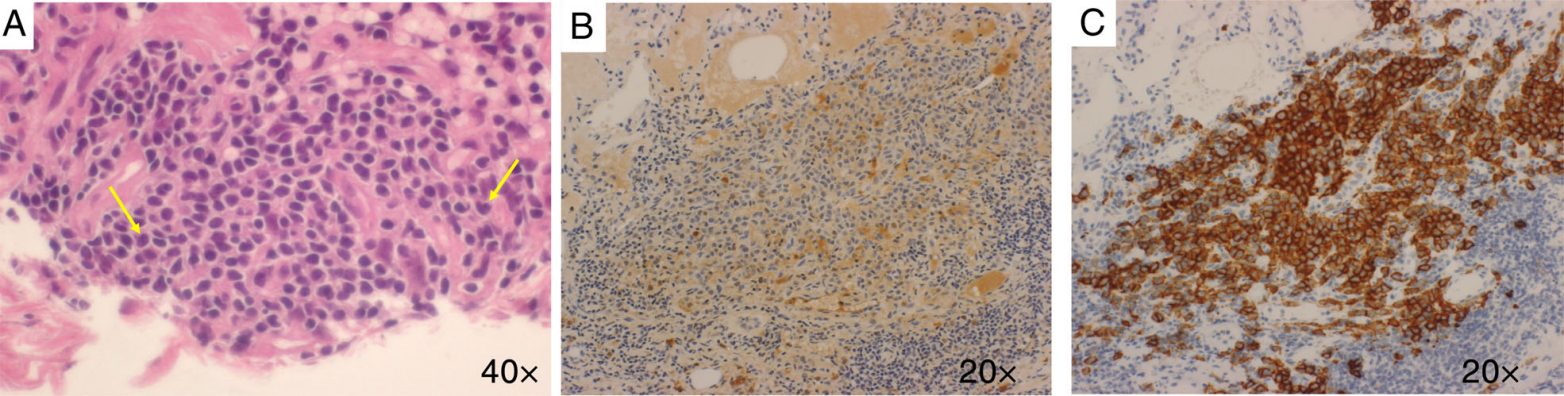
(A–C) Histological findings of transbronchial lung cryobiopsy (TBLC) with histiocytoid cells (haematoxylin–eosin (H–E) stain, 40×). (A) Langerhans cells demonstrate “coffee‐bean”‐shaped grooved nuclei (yellow arrows). Immunohistochemical staining revealed positivity for S‐100 protein (B) and CD1a (C).

A 29‐year‐old non‐smoker initially presented at another facility because of visual field impairment seven years previously. Magnetic resonance imaging (MRI) showed pituitary adenoma. She was managed in an outpatient manner. Cabergoline was started for hyperprolactinaemia. One year later, a decrease in thyroid function was noted. Craniotomy biopsy was considered for diagnostic purposes, but the patient did not consent. One year later, desmopressin was started with the diagnosis of diabetes insipidus. Five years later, at the end of February, she developed pneumonia and was transferred to our hospital. Chest computed tomography (CT) demonstrated multiple thin‐walled cavities and nodules (Fig. 1). Bronchoscopy and transbronchial lung biopsies (TBLB) were performed to evaluate lung findings, however, the results were non‐diagnostic. In August of the same year, TBLC was performed at another hospital. A diagnosis of LCH was finally made based on pathological and immunohistochemical changes (Fig. 2).

Six months before the patient experienced an episode of pneumonia, submandibular gland biopsy was performed at another hospital because of swelling. However, at that time, the biopsy findings only indicated inflammation. We obtained biopsy specimens and performed immunohistochemical analysis, which finally led to the diagnosis of MS‐LCH of the lung and submandibular gland. After the diagnosis, the patient was managed in an outpatient manner and evaluated by forced expiratory volume in 1 sec (FEV_1_), forced vital capacity (FVC), and diffusion capacity for carbon monoxide (DLCO) every three months for more than two years. At two years and two months after diagnosis, she was admitted to our hospital because of difficulty breathing and an increased cyst shadow was noted on the CT scan (Fig. 1). One month before hospitalization, the patient was aware of shortness of breath during exertion. Three months before admission, vulvar ulcers had appeared, and histological findings indicated LCH.

On admission, methylprednisolone pulse therapy (1 g/day for three days) was administered, followed by oral administration of prednisolone (10 mg/day) without tapering. Steroid pulse therapy was repeated at the same dosage after two weeks. During hospitalization, gingivitis was confirmed and LCH was diagnosed histologically. CT findings, arterial partial oxygen pressure, 6‐min walking test, respiratory function test, and dyspnoea during exertion improved after steroid therapy. DLCO had decreased from nearly 50% to 38% before treatment and increased to nearly 70% after treatment. VC was approximately 1.9 L, dropped to 1.6 L on admission, and then improved to 1.7 L one month after treatment. Skin disease and oral mucosal lesions showed improvement, but endocrinopathies did not show any obvious change.

## Discussion

The natural history of PLCH widely varies on an individual basis. Approximately 40–50% of patients experience a favourable outcome, either spontaneously or with glucocorticoid therapy. Partial or complete clearance of radiological abnormalities occurs, and any symptoms resolve. Approximately 10–20% of patients have early severe manifestations, consisting of recurrent pneumothorax or progressive respiratory failure with chronic cor pulmonale. Finally, 30–40% of patients show persistent symptoms of variable severity with conversion of radiological nodules into thick‐walled and then thin‐walled cysts that remain stable over time. Despite the apparent quiescence of the disease in patients with persistent stable cysts, LC granulomas may be present in the pulmonary parenchyma [3].

Thus far, PLCH has been associated with smoking. In particular, smoking status has been associated with the risk of subsequent lung function deterioration as indicated by FEV_1_, FVC, and DLCO [2]. In the present case, the patient was a non‐smoker with MS‐LCH, but her lung lesions worsened within several months. Recent case reports demonstrate that exacerbations of PLCH also occur in non‐smokers [4, 5, 6].

Case reports of non‐smokers with pulmonary lesions that worsened and required treatment are summarized in Table 1. There were four cases including the present case [4, 5, 6]. Two of these cases improved with steroid treatment. One case initially improved with steroid therapy; however, the patient relapsed after seven years of remission and was subsequently treated with methylprednisolone, methotrexate, and irradiation [4]. The other case improved with steroid therapy, which was administered for a few months. There has been no evidence of disease progression during follow‐up [6].

**Table 1:** Case reports of non‐smokers with pulmonary lesions that worsened and required treatment.

Reference	Year	Age (years)/sex	Organs	Therapy
Dubravka [4]	2003	44/Female	Lung	mPSL 0.8 mg/kg and reduction over six months
			After seven years, bones and lung	Weekly mPSL 30 mg/kg i.v. and MTX 50 mg i.v. for six weeks and then 1200 Gy of irradiation in four fractions, and then weekly oral MTX 10 mg for two years
Grobost [5]	2014	37/Female	Lung	Prednisolone 0.5 mg/kg per day for six months and then cladribine i.v. in four courses
			Lung and cranial bone	Three additional courses of cladribine
Jie [6]	2017	38/Male	Lung and bone	Corticosteroid therapy for a few months
Present case	2020	29/Female	Lung, glandula submandibularis, skin, and tooth	mPSL 1 g i.v. biweekly and prednisolone 10 mg p.o.

i.v., Intravenous; mPSL, methylprednisolone; MTX, methotrexate; p.o., per os.

Previous cases have used bronchoscopy, bronchoalveolar lavage fluid, and surgical lung biopsy for the diagnosis of PLCH. To our knowledge, this is the first case in which PLCH was diagnosed by TBLC. Some patients with reduced pulmonary function may be at high risk of perioperative complications following craniotomy biopsy and surgical lung biopsy. Therefore, the usefulness of TBLC is attracting attention in the diagnosis of diffuse lung disease.

In this case, steroid treatment was successful for improvement of symptoms and lung function. In addition, we are actively considering lung transplantation and preparing for transplant registration. Prior publications on adult LCH patients demonstrated little data on the number of relapsed patients, frequency of relapse, or comparative data on responses to different regimens. Thus, we cannot state the risk factors for exacerbations clearly. Systemic therapy should be considered in MS‐LCH patients with risk organs (e.g. bone marrow, liver, spleen, and central nervous system (CNS)). Moreover, there is presently no drug that has been established for adult LCH therapy, unlike paediatric LCH. Treatment with cytarabine, etoposide, vinblastine, or prednisolone has been mentioned in many manuals, but efficacy of these agents has not yet been verified in a prospective study for adult LCH. In this case, we considered the adverse effects of chemotherapy and thus chose steroid therapy. Because LCH is rarely associated with malignant tumours, follow‐up of disease status and monitoring of functional impairment are important. It is recommended that patients are evaluated by FEV_1_, FVC, and DLCO every three to six months [2]. In patients with progressive PLCH, transplantation is the only method to prolong survival [6]. Future accumulation of evidence is expected for adult MS‐LCH cases.

In conclusion, this case study contributes to our understanding of the potential role of steroid therapy in treatment of progressive MS‐LCH. TBLC is a novel diagnostic tool for PLCH.

### Disclosure Statement

Appropriate written informed consent was obtained for publication of this case report and accompanying images.
